# Supplementary material for: A kinetic dissection of the fast and superprocessive kinesin-3 KIF1A reveals a predominant one-head-bound state during its chemomechanical cycle
Source: J Biol Chem. 2021 Jan 13;295(52):17889–903. doi: 10.1074/jbc.RA120.014961 (PMC7939386; doi:10.1074/jbc.RA120.014961)
Supplement: Supplementary file 1 [file mmc1.pdf]

A kinetic dissection of the fast and superprocessive kinesin-3 KIF1A reveals a predominate one-head-bound state during its chemomechanical cycle

Taylor M. Zaniewski, Allison M. Gicking, John Fricks, and William O. Hancock

Supplemental Figures:

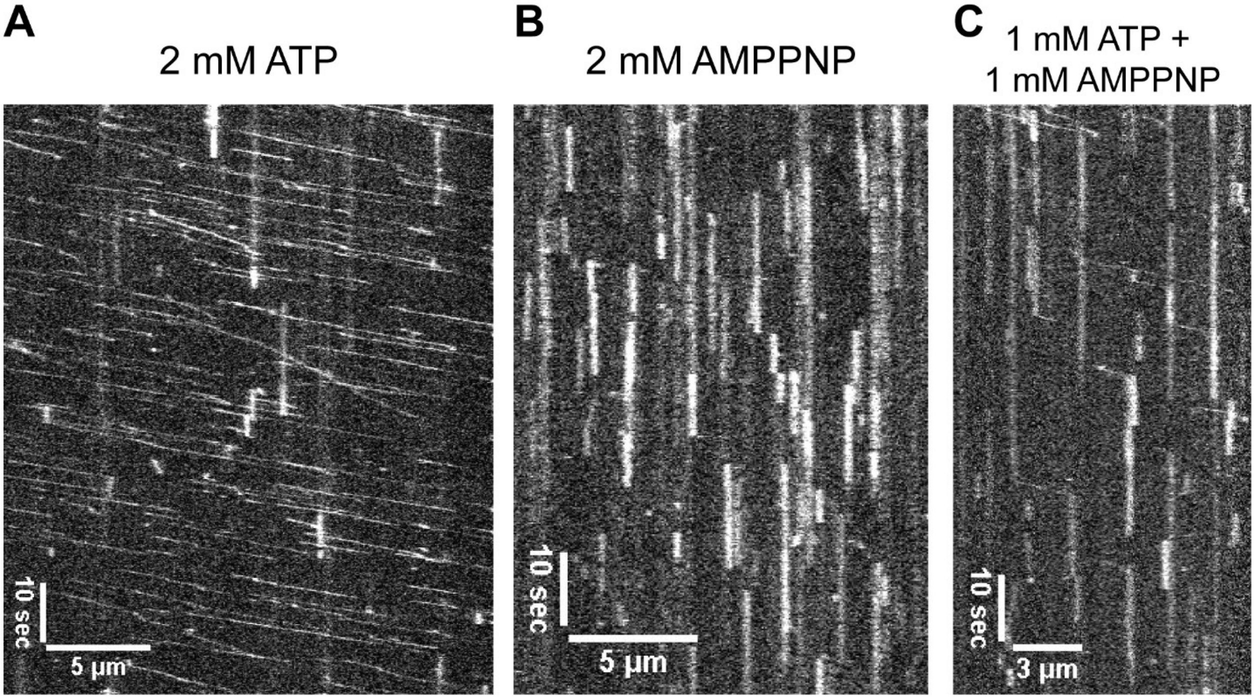

Figure S1. AMPPNP binds to KIF1A and disrupts processive stepping. A, Kymograph of KIF1A-560-GFP in 2 mM ATP at 5 fps. **B,** Kymograph of KIF1A-560-GFP in 2 mM AMPPNP at 5 fps. **C,** Kymograph of KIF1A-560-GFP in 1 mM ATP and 1 mM AMPPNP at 5 fps, showing numerous bound motors and short processive segments interrupted by pauses that confirm that AMPPNP is an ATP analog for KIF1A.

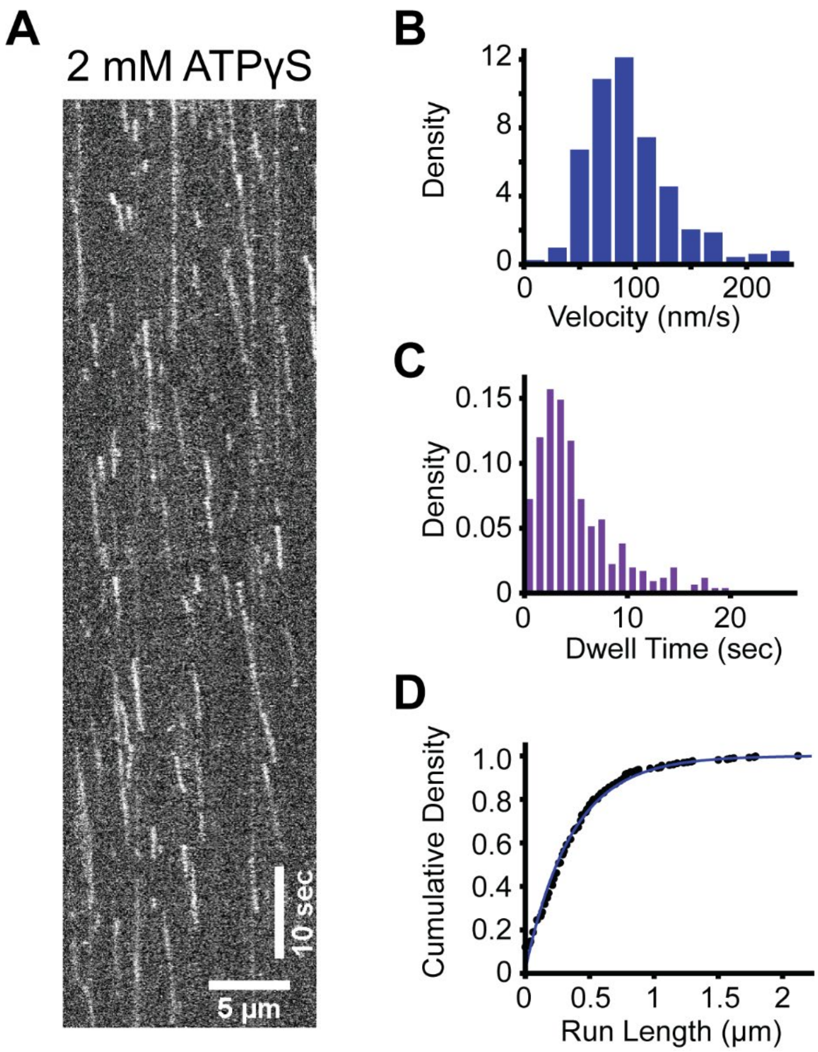

Figure S2. KIF1A steps slowly in ATPγS. A, Kymograph of KIF1A-560-GFP in 2 mM ATPγS at 10 fps. **B,** Velocity distribution of KIF1A-560-GFP in 2 mM ATPγS. Mean velocity was 91.8 ± 2.5 nm/s (Mean ± SEM, N= 278). Population excludes stuck/non-processive and diffusive tracks. **C,** Dwell time distribution of KIF1A-560-GFP in 2 mM ATPγS. Mean duration was 5.3 ± 0.23 s (Mean ± SEM, N = 379). **D,** Run Length distribution of KIF1A-560-GFP in 2 mM ATPγS. Cumulative density fit gives 360 ± 11 nm (fit ± 95% confidence, N = 379).

Supplemental Table:

	Kinesin-1			Kinesin-2			Kinesin-3		
**Step Transition**	**Rate (s^{-1})**	**Duration (s)**	**Percent (%)**	**Rate (s^{-1})**	**Duration (s)**	**Percent (%)**	**Rate (s^{-1})**	**Duration (s)**	**Percent (%)**
**ATP on**	1.2	0.8	<5	18	0.06	<1	1.4	0.7	<8
**Hyd**	281	3.6	<20	478	2.1	<9	na	0	<1
**TH on**	216	4.6	25	117	8.6	35	189	5.3	60
**ADP off**	367	2.7	15	390	2.6	10	354	2.8	32
**RH off**	154	6.5	36	89	11.2	46	na	0	<1
**Total SUM**		**18.2 s**			**24.5 s**			**8.8 s**	
**Stepping**	**533 nm/s**	**15.4 s**		**400 nm/s**	**20.0 s**		**1770 nm/s**	**4.6 s**	

Table S1. Values used to determine the cycle percentages presented in Figure 7. One contribution to the discrepancy between the total sum of durations and the stepping duration for kinesin-3 is the underestimation of the ADP off-rate. This is due to the experiment measuring the unstrained off-rate, rather than the strained rate, which is expected to be faster and hence the duration shorter. Kinesin-1 and -2 values are from Mickolajczyk and Hancock 2017 (28), Mickolajczyk *et al*. 2015 (27), and Chen *et al.* 2015 (34).
