# Supplementary material for: A kinetic dissection of the fast and superprocessive kinesin-3 KIF1A reveals a predominant one-head-bound state during its chemomechanical cycle
Source: J Biol Chem. 2021 Jan 13;295(52):17889–903. doi: 10.1074/jbc.RA120.014961 (PMC7939386; doi:10.1074/jbc.RA120.014961)
Supplement: Supplementary file 2 — Taylor M. Zaniewski [file mmc2.pdf]

Taylor M. Zaniewski

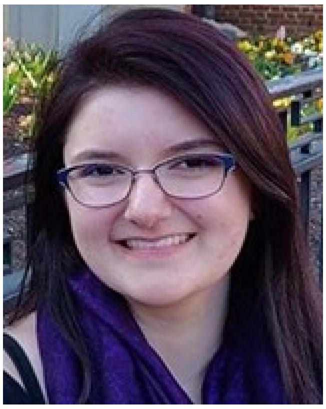

Current position: Graduate Student, Department of Chemistry, Pennsylvania State University, University Park, Pennsylvania, USA

Education: B.S. in Biochemistry, 2017, Central Connecticut State University, New Britain, Connecticut, USA

Where to find me: https://sites.psu.edu/hancocklab/; https://twitter.com/TayMae135

Can you describe an exciting moment you experienced while doing this research?

The most satisfying feeling came from piecing together the puzzle. I had been collecting data and learning so many new things about my protein, but the complete model was still open-ended. The most exciting moment was when it all finally clicked, and it painted a full picture, because it felt like I had the answers to all my questions.

If you could go back in time and re-do this project, what advice would you give your past self?

I would say to learn the fundamental behaviors of your protein before characterizing the more interesting traits. One such example is to determine the affinity of the protein for the nucleotide before performing more intensive experiments to ensure complete labeling and accurate concentration determination. This will allow you to avoid artifacts in your results and save time overall.

What do you hope to do next?

My next steps are to establish myself as a biochemical scientist in industry. I hope to continue to advance health and medical fields through the work of fundamental research.

Read Zaniewski's article on page 17889.
